# Association of Oral Papivir/Pavirona^®^ Supplementation with HPV DNA Clearance

**DOI:** 10.3390/v18040455

**Published:** 2026-04-09

**Authors:** Betul Gungor Serin, Bilal Esat Temiz, Haticegul Tuncer, Muhammed Onur Atakul, Ali Can Gunes, Taylan Onat, Utku Akgor, Derman Basaran, Zafer Selcuk Tuncer, Murat Gultekin

**Affiliations:** 1 Department of Obstetrics and Gynecology, Hacettepe University, Ankara 06230, Turkey; betulgungorserin@gmail.com (B.G.S.); drhaticegultuncer@gmail.com (H.T.); onuratakul@hacettepe.edu.tr (M.O.A.); dr.acgunes@gmail.com (A.C.G.); 2 Department of Obstetrics and Gynecology, Division of Gynecologic Oncology, Hacettepe University, Ankara 06230, Turkey; bilalesattemiz@gmail.com (B.E.T.); utkuakgor@gmail.com (U.A.); dermanbasaran@gmail.com (D.B.); zstuncer@hacettepe.edu.tr (Z.S.T.); 3 Sincan Training and Research Hospital, Ankara 06930, Turkey; taylan.onat@sbu.edu.tr

**Keywords:** human papillomavirus, HPV DNA clearance, oral supplementation, cervical cytology, conservative management

## Abstract

Background: Persistent cervical human papillomavirus (Human papillomavirus) infection remains a significant public health concern, as it is the primary etiological factor in the development of cervical cancer and its precursor lesions. While prophylactic vaccination and standard screening programs are cornerstones of prevention, a substantial proportion of women with established infection are managed conservatively, often with prolonged follow-up and associated psychological burden. Interest has therefore grown in supportive interventions that may facilitate viral clearance during routine clinical management. Methods: This retrospective cohort study included 239 women with confirmed cervical Human papillomavirus infection followed at a tertiary referral center between February 2023 and August 2025. Participants were classified into a treatment group receiving oral Papivir/Pavirona^®^ twice daily for six months (*n* = 119) and a control group managed with routine clinical follow-up alone (*n* = 120). Human papillomavirus DNA testing and cervical cytology were evaluated at baseline and at 6 and 12 months. Results: Human papillomavirus clearance rates were significantly higher in the Papivir/Pavirona^®^ group compared with controls at both 6 and 12 months. Cytological regression was also more frequent in the treatment group at both time points. In multivariate logistic regression analysis, Papivir/Pavirona^®^ use emerged as the only independent predictor of both Human papillomavirus clearance and cytological regression, while demographic, reproductive, behavioral, and virological baseline characteristics were not significantly associated with outcomes. Conclusions: Papivir/Pavirona^®^ supplementation was associated with increased Human papillomavirus clearance and cytological regression rates in women with cervical Human papillomavirus infection, suggesting a potential supportive role alongside standard clinical follow-up.

## 1. Introduction

Cervical cancer is a major global public health problem and continues to be one of the most common malignancies affecting women worldwide. Despite being largely preventable, it remains a leading cause of cancer-related morbidity and mortality, particularly in low- and middle-income countries, where access to effective screening, vaccination and treatment programs is limited [1]. According to recent global estimates, cervical cancer ranks as the fourth most commonly diagnosed cancer and the fourth leading cause of cancer-related death among women worldwide [2].

Persistent infection with human papillomavirus (HPV) is widely recognized as the main etiological factor in cervical cancer development [3]. HPV is a sexually transmitted virus, with more than 200 identified genotypes, approximately 13 of which are classified as high-risk due to their oncogenic potential. Among these, HPV16 and HPV18 are the most clinically significant, accounting for nearly 70% of cervical cancer cases worldwide, while other high-risk HPV types contribute primarily to cervical intraepithelial neoplasia and a smaller proportion of invasive cancers, reflecting the heterogeneity of HPV-related disease [4,5,6].

HPV is transmitted primarily through sexual contact [7]. Although approximately 70–90% of HPV infections are transient and clear spontaneously within one to two years, often within the first 6–12 months depending on viral genotype, persistent infection with high-risk HPV types may lead to premalignant cervical epithelial changes and progression to cervical intraepithelial neoplasia [8,9,10,11]. Accordingly, persistence of infection rather than initial exposure represents the key determinant of progression toward invasive cervical cancer [12].

At the cellular level, persistent high-risk HPV infection drives cervical carcinogenesis primarily through the oncoproteins E6 and E7. HPV E6 forms a trimeric complex with the cellular ubiquitin ligase E6AP and p53, targeting p53 for proteasomal degradation, thereby suppressing apoptosis and enabling unrestricted cell proliferation [13,14]. Similarly, E7 targets the retinoblastoma protein pRb for degradation, driving uncontrolled cell cycle progression. Beyond these well-known mechanisms, E6 and E7 activate the NF-κB signaling pathway, leading to upregulation of pro-inflammatory cytokines and creation of an inflammatory tumor microenvironment that is particularly pronounced in high-grade cervical lesions [15,16]. Concurrently, HPV oncoproteins stabilize hypoxia-inducible factor 1-alpha (HIF-1α) even under normoxic conditions, which drives reactive oxygen species (ROS) production and further amplifies NF-κB-mediated inflammation, collectively facilitating disease progression and immune evasion [17,18].

Prophylactic HPV vaccination is highly effective in preventing new HPV infections and HPV-related precancerous lesions when administered prior to sexual debut. However, vaccination does not reliably eliminate established HPV infections nor consistently accelerate viral clearance in women who are already HPV-positive. Moreover, in many countries, HPV vaccination programs are newly implemented, incompletely established, or limited by socioeconomic and structural barriers. Therefore, a substantial proportion of women worldwide remain unvaccinated or only partially protected, and HPV infection continues to pose a major clinical challenge [19,20].

In routine clinical practice, HPV-positive women, particularly those with normal cytology or low-grade abnormalities, are commonly managed with standard clinical follow-up, involving repeated HPV testing and cervical cytology at defined intervals [21]. Although this approach is evidence-based and aligns with international guidelines, it often requires prolonged follow-up and may be associated with considerable psychological burden, including anxiety, fear of cancer development, and uncertainty regarding disease progression [22]. Consequently, interest has increased in supportive, non-invasive options that may support HPV clearance during routine follow-up.

In recent years, attention has increasingly focused on nutraceutical and phytochemical agents that can be administered orally and may exert antiviral and antioxidant effects. A comprehensive review published in 2024 highlighted that a range of supportive agents, some available by physician prescription, are being explored as adjunctive approaches in gynecological cancer prevention [23]. In parallel with this growing interest, clinical studies investigating HPV clearance and cytological regression associated with such agents have gained momentum over the past decade [24,25,26,27,28,29].

Papivir/Pavirona^®^ (Mealis, Istanbul, Türkiye) is an oral, multi-component formulation containing five bioactive compounds such as quercetin, selenium, cinnamon extract, green tea extract (catechins) and licorice root extract, whose antiviral, antioxidant, and cell-regulatory effects relevant to HPV persistence have largely been demonstrated in experimental models. One of these components, quercetin, has been extensively evaluated in vitro and has been shown to induce cell-cycle arrest and apoptosis in HPV-positive cervical cells through p53-related pathways [30,31]. In parallel, experimental studies have shown that cinnamon extract exerts antiproliferative effects in HPV-positive cervical cell models, with increased apoptotic activity and suppression of NF-κB-related signaling pathways involved in HPV-related cellular change [32,33]. Similarly, glycyrrhizin, the principal bioactive component of licorice root extract, has likewise been evaluated in HPV-positive cervical epithelial cell models. Experimental studies have shown that glycyrrhizin induces cell-cycle arrest and apoptosis, accompanied by downregulation of HPV E6/E7 oncogene expression and attenuation of proliferative signaling pathways [34,35]. Selenium, another component, has been evaluated in both experimental and clinical studies. Experimental evidence suggests that selenium supports cellular antioxidant defense and redox balance through modulation of ROS levels and selenoprotein activity [36,37], while observational and clinical studies have reported lower serum selenium levels in women with cervical intraepithelial neoplasia compared to cytologically normal controls, suggesting a potential association with cervical dysplasia [38].

As the final component of the formulation, green tea catechins, particularly epigallocatechin gallate (EGCG), have been evaluated in experimental studies related to HPV-associated cervical disease. In vitro studies have shown that EGCG can inhibit HPV-related oncogenic activity and induce apoptosis in HPV-positive cervical epithelial cells [39,40], supporting its role as part of a multi-component formulation.

However, although preliminary studies have suggested potential benefits [25], the clinical evidence regarding the effect of this specific formulation on HPV DNA clearance and cervical cytological outcomes remains limited and not yet clearly established.

Therefore, the aim of the present study was to assess the association between Papivir/Pavirona^®^ supplementation and HPV DNA clearance as well as cervical cytological regression in women with confirmed HPV infection managed according to standard clinical guidelines. We hypothesized that oral Papivir/Pavirona^®^ supplementation, through its combined antiviral, antioxidant, and immunomodulatory properties, would be associated with higher rates of HPV DNA clearance and cytological regression compared with standard clinical follow-up alone.

## 2. Materials and Methods

### 2.1. Study Design and Participants

This retrospective cohort study was conducted at the Department of Obstetrics and Gynecology, Hacettepe University Hospital, Ankara, Türkiye, covering the period between January 2023 and August 2025. Patient demographic and clinical data were obtained from archived medical records, including patient follow-up files, hospital archive records, hospital procedural databases, and comprehensive medical information files encompassing follow-up data from other healthcare centers.

A total of 239 patients with a documented positive human papillomavirus (HPV) deoxyribonucleic acid (DNA) test who also had baseline cervical cytology results were included in the study. Cytological findings included negative for intraepithelial lesion or malignancy (NILM), atypical squamous cells of undetermined significance (ASC-US), atypical squamous cells—cannot exclude high-grade squamous intraepithelial lesion (ASC-H), low-grade squamous intraepithelial lesion (LSIL), high-grade squamous intraepithelial lesion (HSIL) or atypical glandular cells (AGC). Patients were excluded from the study if medical records indicated the presence of sexually transmitted infections or symptomatic vulvovaginal infections, immunodeficiency or autoimmune diseases, use of immunosuppressive therapy, pregnancy, total hysterectomy, or a history of gynecologic malignancy.

Patients were classified into two groups based on routine clinical management documented in their medical records. The treatment group consisted of women who had been prescribed oral Papivir/Pavirona^®^ tablets twice daily as part of their clinical care. In this group, Papivir/Pavirona^®^ supplementation was administered continuously for a total duration of six months. The control group included women who were managed with standard clinical follow-up without any adjunctive pharmacological or nutraceutical intervention. As part of routine counseling, patients were advised regarding safe sexual practices; condom use was recommended, although sexual intercourse was not restricted. Patients were routinely counseled to avoid vaginal douching and the use of intravaginal deodorant products during the follow-up period. HPV DNA testing and cervical cytology assessments performed at baseline and during routine follow-up visits at approximately 6 and 12 months were retrieved from patient records for analysis.

Use of Papivir/Pavirona^®^ was documented in medical records as an adjunctive supplement and was not intended to influence routine clinical management or standard-of-care decision-making. Diagnostic and therapeutic procedures, including colposcopy, directed cervical biopsy, and excisional treatments such as loop electrosurgical excision procedure (LEEP), were performed based on clinical indications and in accordance with established cervical cancer screening and management guidelines [21]. Papivir/Pavirona^®^ use was therefore recorded as a supportive supplementation and did not alter or delay diagnostic or therapeutic decisions within routine clinical practice.

### 2.2. Ethics

The study received approval from the Ministry of Health Local Ethics Committee (Approval No: SEAH-BAEK-2025-151) and was conducted in accordance with the principles out-lined in the Declaration of Helsinki.

### 2.3. Statistical Analysis

The primary endpoint of the study was HPV DNA clearance at 6 and 12 months. Secondary endpoints included cytological regression, high-risk HPV clearance, and treatment safety and tolerability outcomes. Data regarding HPV DNA testing and cervical cytology (Papanicolaou test), which were performed as part of routine clinical practice, were retrospectively retrieved from medical records at baseline and during follow-up visits at approximately 6 and 12 months. According to standard clinical procedures, exocervical and endocervical cells had been collected using a cytobrush and sent to the Department of Pathology for examination. Cytological results were classified according to the 2001 Bethesda System as negative for intraepithelial lesion or malignancy, ASC-US, ASC-H, AGC, LSIL, or HSIL [41].

For HPV testing, cervical specimens were collected using a sterile Copan eSwab^®^ collection and preservation system. After collection, the swab was placed into the transport medium and stored at room temperature until further processing. Cervical cells were subsequently suspended in PreservCyt solution (Hologic, Marlborough, MA, USA) and stored at room temperature until analysis. DNA extraction was performed using an eMAG automated extractor (bioMérieux, Marcy l’Etoile, France), with samples resuspended in 1 mL of buffer according to the manufacturer’s instructions. HPV DNA detection and genotyping were conducted using the Anyplex™ II HPV28 real-time PCR assay (Seegene, Seoul, South Korea).

HPV clearance was evaluated by considering both complete and partial resolution of infection over time. Complete clearance was defined as a negative HPV DNA test or the disappearance of all HPV genotypes identified at baseline. Partial clearance referred to the disappearance of at least one initially detected HPV genotype. Smear clearance was defined using the same classification as HPV clearance. Overall smear clearance was defined as the occurrence of either total or partial clearance. Total smear clearance was defined as the complete resolution of the baseline cytological abnormality with a return to normal cervical cytology. Partial smear clearance was defined as regression from the baseline cervical lesion to a lower-grade cervical lesion. Patients with normal or non-malignant baseline cytology were excluded from the cytological outcome analysis. Clinical, cytological, colposcopic and laboratory data were routinely collected during standard follow-up visits and assessed at baseline, as well as at 6 and 12 months.

For methodological analysis, HPV genotypes were categorized into five groups: HPV 16, HPV 18, other high-risk HPV types (HPV 31, 33, 35, 39, 45, 51, 52, 56, 58, 59, 68, 73, and 82), intermediate-risk HPV types (HPV 26, 53, and 66), and low-risk HPV types (HPV 6, 11, 40, 42, 43, 44, 54, 61, 70, 72, and 81).

Data were analyzed using IBM SPSS version 23. The conformity of continuous variables to a normal distribution was assessed using the Kolmogorov–Smirnov and Shapiro–Wilk tests. Associations between categorical variables were examined using the chi-square test. The effects of independent variables on HPV and cytological regression responses were evaluated using binary logistic regression analysis. Results were presented as mean ± standard deviation for quantitative data and as frequency (percentage) for categorical variables. A *p*-value of <0.05 was considered statistically significant.

## 3. Results

### 3.1. Study Population and Baseline Characteristics

There were 239 women included in the study. Of these, 119 participants (49.8%) received Papivir/Pavirona^®^, while 120 participants (50.2%) were managed in the control group. Baseline demographic and clinical characteristics of both groups are presented in Table 1.

The mean age was 35.03 ± 9.28 years in the Papivir/Pavirona^®^ group and 37.01 ± 8.26 years in the control group, with no statistically significant difference between groups (*p* = 0.082). Age group distribution (<30, 30–45, and >45 years) was also comparable between the two groups (*p* = 0.180).

Baseline HPV genotype distribution was similar in Papivir/Pavirona^®^ users and controls. The proportions of HPV16, HPV18, other high-risk, intermediate-risk, and low-risk HPV types did not differ significantly between groups (*p* = 0.540).

Baseline cervical cytology findings were likewise comparable between groups. The distribution of normal cytology, atypical squamous cells of undetermined significance (ASC-US), atypical squamous cells—cannot exclude HSIL (ASC-H), low-grade squamous intraepithelial lesion (LSIL), high-grade squamous intraepithelial lesion (HSIL), and atypical glandular cells (AGC) showed no statistically significant difference between the Papivir/Pavirona^®^ and control groups (*p* = 0.270).

Overall, the two groups were well balanced at baseline with respect to age, HPV genotype distribution, and cervical cytological status, allowing for a valid comparison of follow-up outcomes.

Among women using Papivir/Pavirona^®^, loop electrosurgical excision procedure (LEEP) was performed in 11 of 16 patients with HSIL, 4 of 8 patients with ASC-H, 3 of 20 patients with LSIL, and in one patient with atypical glandular cells (AGC). In the control group, LEEP was applied in 6 of 8 patients with HSIL, 7 of 23 patients with LSIL, 2 of 5 patients with ASC-H, and 5 of 26 patients with ASC-US. Histopathological examination of LEEP specimens revealed cervicitis in 3 patients (15.8%) in the treatment group and 2 patients (13.3%) in the control group. CIN 1 was identified in 4 Papivir/Pavirona^®^ treated patients (21.1%) and 3 control patients (20.0%), CIN 2 in 3 (15.8%) and 4 (26.7%), and CIN 3 in 9 (47.4%) and 6 (40.0%) patients, respectively.

### 3.2. HPV Clearance and Cytological Regression Outcomes

As shown in Table 2, HPV clearance and cytological regression rates at 6 months were significantly higher in the Papivir/Pavirona^®^ group than in the control group, despite similar baseline characteristics. Overall HPV clearance was achieved in 76.5% of Papivir/Pavirona^®^ users compared with 47.5% of controls at 6 months (*p* < 0.001), with complete clearance rates of 65.5% versus 29.2% and partial clearance rates of 10.9% versus 18.3%, respectively. Among HPV genotype subgroups, complete clearance rates were consistently higher in the Papivir/Pavirona^®^ group across all categories, including HPV 16 (68.2% vs. 27.5%), HPV 18 (75.0% vs. 12.5%), other high-risk types (60.0% vs. 35.3%), intermediate-risk types (66.7% vs. 21.4%), and low-risk types (69.2% vs. 28.6%). Regarding cytological outcomes, overall regression was observed in 83.8% of Papivir/Pavirona^®^ users compared with 70.0% of controls (*p* = 0.012), with complete regression rates of 81.1% versus 58.3% and partial regression rates of 2.7% versus 11.7%, respectively. Complete cytological regression was higher in the Papivir/Pavirona^®^ group across all cytological subgroups, including ASC-US (72.4% vs. 57.7%), ASC-H (87.5% vs. 40.0%), LSIL (90.0% vs. 60.9%), and HSIL (81.3% vs. 75.0%).

Twelve-month follow-up data were available for 94 patients in the Papivir/Pavirona^®^ group and 100 patients in the control group. Follow-up data at 12 months could not be obtained for 25 patients in the treatment group and 20 patients in the control group.

At 12 months, in the Papivir/Pavirona^®^ group, complete HPV clearance was observed in 65 patients (69.1%), partial clearance in 11 patients (11.7%), and no clearance in 18 patients (19.1%). In the control group, complete clearance occurred in 46 patients (46.0%), partial clearance in 10 patients (10.0%), and no clearance in 44 patients (44.0%) (*p* = 0.001).

At 12 months, in the Papivir/Pavirona^®^ group, complete cytological regression was observed in 53 patients (77.9%), partial regression in 5 patients (7.4%), and persistent cytological abnormality in 10 patients (14.7%). In the control group, complete regression occurred in 19 patients (52.8%), partial regression in 3 patients (8.3%), and persistent abnormality in 14 patients (38.9%) (*p* = 0.017).

### 3.3. Multivariable Logistic Regression Analysis

Values are presented as row percentages. Univariate logistic regression analyses were performed to assess the association between each variable and HPV clearance. Multivariable logistic regression analysis was performed in the combined cohort. OR: odds ratio; aOR: adjusted odds ratio; CI: confidence. Univariate and multivariate logistic regression analyses were performed to identify factors associated with HPV clearance at 6 months in the combined cohort (Table 3).

In univariate analysis, Papivir/Pavirona^®^ use was significantly associated with HPV clearance. Clearance was observed in 76.5% of Papivir/Pavirona^®^ users compared with 47.5% of non-users, corresponding to a higher likelihood of HPV clearance among users (OR = 3.59, 95% CI: 2.06–6.26; *p* < 0.001).

After adjustment for potential confounders in the multivariate model, Papivir/Pavirona^®^ use remained the only independent predictor of HPV clearance. Participants using Papivir/Pavirona^®^ had nearly a fivefold higher likelihood of achieving HPV clearance compared with non-users (adjusted OR = 4.80, 95% CI: 2.26–10.17; *p* < 0.001).

None of the other evaluated variables demonstrated a statistically significant association with HPV clearance in either univariate or multivariate analyses. Age group, pregnancy status, menstrual status, oral contraceptive use, smoking, history of LEEP, HPV vaccination status, and baseline HPV genotype distribution were not significantly associated with clearance outcomes (all *p* > 0.05).

Review of medical records identified one patient who developed a mild cutaneous rash during Papivir/Pavirona^®^ use, which resolved spontaneously without discontinuation of treatment. No other adverse events related to Papivir/Pavirona^®^ were documented during the follow-up period.

## 4. Discussion

In this retrospective cohort study, outcomes of women with cervical HPV infection who received Papivir/Pavirona^®^ supplementation for six months were evaluated during a 12-month follow-up period and compared with those managed with standard clinical follow-up alone at a tertiary referral center. Papivir/Pavirona^®^ use was associated with higher rates of HPV DNA clearance and cytological regression compared with non-users throughout follow-up.

Specifically, HPV clearance and cytological regression occurred at approximately twofold higher rates among patients receiving Papivir/Pavirona^®^, and this association remained independent of age group and other demographic, reproductive, and behavioral variables included in the analysis. Notably, Papivir/Pavirona^®^ use emerged as the only independent predictor of both virological and cytological improvement in multivariate models.

Taken together, these findings suggest that orally administered, multi-component phytochemical formulations may support HPV regression during routine clinical follow-up by combining complementary antiviral and antioxidant activities.

The observed differences in HPV clearance and cytological regression rates between the Papivir/Pavirona^®^ and control groups may be partly explained by the molecular mechanisms of its bioactive components, as outlined in the Introduction. Specifically, the NF-κB inhibitory properties of quercetin and cinnamon extract may help attenuate the pro-inflammatory tumor microenvironment that sustains HPV persistence in high-grade cervical lesions [15,30,31,33]. Simultaneously, the antioxidant effects of selenium and green tea catechins may counteract HPV-driven ROS production and oxidative stress, thereby reducing conditions favorable to viral persistence [36,40]. Furthermore, the potential modulation of HIF-1α stabilization by these compounds may help disrupt the hypoxia-driven signaling cascade that facilitates immune evasion and disease progression in HPV-positive cervical epithelium [17,18]. Additionally, quercetin has been shown to induce p53-dependent apoptosis and cell cycle arrest in HPV-positive cervical cells through disruption of the E6/E6AP/p53 complex, suggesting a potential mechanistic basis for p53 pathway restoration in HPV-infected epithelium [30,42]. Taken together, the multi-targeted nature of Papivir/Pavirona^®^, simultaneously addressing inflammatory, oxidative, and hypoxic pathways, may underlie the consistently higher clearance rates observed across different HPV genotype categories and cytological grades in the present study.

Beyond its virological and cytological effects, the potential clinical implications of Papivir/Pavirona^®^ supplementation extend to the psychological and quality-of-life dimension of HPV management. Women with persistent HPV infection, particularly those with normal cytology or low-grade abnormalities, are commonly managed with watchful waiting and repeated testing over extended periods. This prolonged follow-up is frequently associated with significant psychological burden, including anxiety, fear of cancer progression, and uncertainty regarding disease outcomes. In this context, a well-tolerated oral supplement that may support viral clearance during routine follow-up could represent a meaningful adjunctive option, particularly for patients who prefer non-invasive management strategies or those requiring prolonged surveillance. The favorable safety profile observed in the present study, with only one case of mild cutaneous rash reported during the follow-up period, further supports the tolerability of this formulation in clinical practice.

In the present study, smoking status was not significantly associated with HPV clearance in either univariate or multivariate analyses. However, cigarette smoking is well established as one of the most important cofactors in cervical carcinogenesis, independent of HPV infection [43]. Proposed mechanisms include smoking-induced local immunosuppression through reduction in Langerhans cell density, impaired T-cell mediated immune surveillance, and direct genotoxic effects of tobacco carcinogens in cervical mucus [44]. Furthermore, tobacco use has been associated with increased risk of persistent HPV infection [45]. The absence of a significant association in the present cohort may reflect the relatively small proportion of smokers in the study population and limited statistical power for subgroup analyses, rather than a true absence of effect.

Only one earlier clinical study, aside from the present analysis, has evaluated a formulation with the same bioactive composition. Gene-Eden-VIR/Novirin^®^, an oral supplement composed of the same five active components as Papivir/Pavirona^®^ in identical proportions, has previously been evaluated in a single post-marketing clinical study. In that study, 139 HPV-positive individuals received oral supplementation for variable durations ranging from 2 to 12 months, and outcomes were assessed based on changes in HPV persistence rather than fixed clearance rates. The authors reported a shortening of HPV persistence during treatment and a favorable safety profile across different age groups and HPV genotypes. Although the study design and outcome measures differed from those of the present cohort, these findings provide complementary clinical evidence supporting the potential role of this multi-component formulation in the management of HPV infection and represent the only other published clinical evaluation of this specific compound combination [25].

A key strength of this study is the evaluation of patient outcomes within routine clinical practice, with management decisions following standard gynecological guidelines and made independently of research participation. The consistent use of HPV DNA testing and cervical cytology at baseline and follow-up enabled reliable assessment of changes over time, while inclusion of a contemporaneous control group provided a meaningful clinical reference for interpreting clearance and regression outcomes.

Several limitations should be acknowledged. As a single-center study, the findings may not be fully generalizable to other populations or healthcare settings. In addition, the retrospective design and reliance on routinely collected clinical data limited the availability of detailed information on treatment adherence and lifestyle factors that may influence HPV clearance. While patients with no documented history of prior HPV-related treatment in their medical records were included in the analysis, the possibility that some patients may have received HPV-related interventions at other healthcare facilities prior to their referral to our center cannot be excluded, as external medical records were not systematically available. Additionally, information regarding the presence or absence of a regular sexual partner and HPV testing status of sexual partners was not available in the clinical records, which may represent an additional confounding factor in the interpretation of HPV clearance outcomes. Finally, as HPV viral load results were not available for the study population, the potential influence of viral load on clearance outcomes could not be assessed, which represents a further limitation of the present study.

Despite these limitations, the findings of the present study have relevant implications for clinical practice and future research. The consistently higher rates of HPV DNA clearance and cytological improvement observed among patients receiving Papivir/Pavirona^®^ suggest that this oral, multi-component formulation may represent a supportive option alongside standard clinical follow-up in conservatively managed HPV-positive patients. This may be beneficial for patients requiring prolonged follow-up, particularly when non-invasive strategies are favored. Future studies employing prospective, randomized controlled designs with longer follow-up are warranted to confirm the durability of these effects, evaluate recurrence rates, and further define patient subgroups most likely to benefit from supplementation.

## 5. Conclusions

Collectively, the findings of this retrospective cohort study indicate that oral Papivir/Pavirona^®^ supplementation was associated with significantly higher rates of HPV DNA clearance and cytological regression compared with standard clinical follow-up alone. These findings are consistent with our initial hypothesis, supporting the notion that the combined antiviral, antioxidant, and immunomodulatory properties of Papivir/Pavirona^®^, acting through multiple molecular pathways including NF-κB inhibition, attenuation of reactive oxygen species production, modulation of HIF-1α stabilization, and partial restoration of p53-mediated tumor suppressor activity, may contribute to enhanced HPV clearance and cytological regression during conservative management. The consistently higher clearance rates observed across different HPV genotype categories and cytological grades suggest a broad-spectrum adjunctive benefit of this multi-component formulation. From a clinical perspective, these findings may have implications for the management of HPV-positive women undergoing prolonged conservative follow-up, particularly those seeking non-invasive supportive options alongside standard care. At the same time, the present data should not be viewed as definitive, and future prospective, randomized controlled studies are warranted to confirm these findings and further elucidate the mechanisms underlying the observed clinical benefit.

## Figures and Tables

**Table 1:** Distribution of age, HPV genotype positivity and Pap smear cytology between study groups.

	Papivir (*n* = 119)	Control (*n* = 120)	*p*-Value
**Age (years), mean ± SD**			0.082
	35.03 ± 9.28	37.01 ± 8.26	
**Age group (years)**			0.180
<30	35 (29.4%)	24 (20.0%)	
30-45	65 (54.6%)	76 (63.3%)	
>45	19 (16.0%)	20 (16.7%)	
**HPV Positivity by Genotype**			***p*** **-value**
			0.540
HPV 16	44 (37.0%)	40 (33.3%)	
HPV 18	8 (6.7%)	8 (6.7%)	
Other High-Risk HPV Types	45 (37.8%)	51 (42.5%)	
Intermediate-Risk HPV Types	9 (7.6%)	14 (11.7%)	
Low-Risk HPV Types	13 (10.9%)	7 (5.8%)	
	**Papivir (*n* = 119)**	**Control (*n* = 120)**	***p*** **-value**
**Pap Smear Cytology**			0.270
Normal Cytology	45 (37.8%)	58 (48.3%)	
ASC-US	29 (24.4%)	26 (21.7%)	
ASC-H	8 (6.7%)	5 (4.2%)	
LSIL	20 (16.8%)	23 (19.2%)	
HSIL	16 (13.4%)	8 (6.7%)	
AGC	1 (0.8%)	0 (0%)	

Data are presented as mean ± standard deviation or *n* (%), as appropriate. Percentages are calculated based on valid cases. Patients may have more than one HPV genotype.

**Table 2:** Association Between Papivir/Pavirona^®^ Use and HPV Clearence and Cytological Regression at 6 Months.

	CLEARANCE				
	**Papivir (*n* = 119)**		**Control (*n* = 120)**		
	**Yes**	**No**	**Yes**	**No**	***p*** **-value**
**HPV Genotype**					
HPV 16	32 (72.7%)	12 (27.3%)	19 (47.5%)	21 (52.5%)	
HPV 18	7 (87.5%)	1 (12.5%)	5 (62.5%)	3 (37.5%)	
Other High-Risk Types	34 (75.6%)	11 (24.4%)	27 (52.9%)	24 (47.1%)	
Intermediate-Risk Types	7 (77.8%)	2 (22.2%)	4 (28.6%)	10 (71.4%)	
Low-Risk types	11 (84.6%)	2 (15.4%)	2 (28.6%)	5 (71.4%)	
Total	91 (76.5%)	28 (23.5%)	57 (47.5%)	63 (52.5%)	<0.001
	**Papivir (*n* = 119)**		**Control (*n* = 120)**		
**Pap Smear Cytology**	**Yes**	**No**	**Yes**	**No**	***p*** **-value**
ASC-US	21 (72.4%)	8 (26.7%)	15 (57.7%)	11 (42.3%)	
ASC-H	7 (87.5%)	1 (12.5%)	4 (80.0%)	1 (20.0%)	
LSIL	18 (90.0%)	2 (10.0%)	17 (82.6%)	6 (26.1%)	
HSIL	15 (93.8%)	1 (6.2%)	8 (100.0%)	0 (0%)	
AGC	1 (100%)	0 (0%)	0 (0%)	0 (0%)	
Total	62 (83.8%)	12 (16.2%)	44 (70.0%)	18 (30.0%)	0.012

*p*-values refer to comparisons of overall clearance between the Papivir/Pavirona^®^ and control groups (Chi-square test). Values are presented as row percentages.

**Table 3:** Binary logistic regression analysis of the effect of independent variables on HPV clearance at 6 months.

	Clearance (+), *n* (%)	Clearance (−), *n* (%)			Combined Cohort	
Variable			Univariate OR (95% CI)	*p*-Value	Multivariate aOR (95% CI)	*p*-Value
**Papivir Use**						
No	57 (47.5%)	63 (52.5%)	Reference	<0.001	4.80 (2.26–10.17)	<0.001
Yes	91 (76.5%)	28 (23.5%)	3.59 (2.06–6.26)			
**Age**				0.936		
<30	34 (57.6%)	25 (42.4%)	Reference		1.72 (0.84–3.55)	0.137
30–45	92 (65.2%)	49 (34.8%)	1.05 (0.46–2.38)	0.905		
>45	22 (56.4%)	17 (43.6%)	1.45 (0.70–3.00)	0.312		
**Pregnancy**						
Nulliparous	60 (68.2%)	28 (31.8%)	Reference		0.64 (0.29–1.40)	0.262
Multiparous	55 (62.5%)	33 (37.5%)	1.29 (0.69–2.40)	0.429		
**Menstrual Status**						
Regular	102 (67.5%)	49 (32.5%)	Reference		0.52 (0.25–1.05)	0.076
Irregular	8 (53.3%)	7 (46.7%)	1.49 (0.45–4.94)	0.516		
Menopause	7 (58.3%)	5 (41.7%)	0.82 (0.18–3.66)	0.795		
**Oral Contraceptive Use**						
No	102 (61.4%)	64 (38.6%)	Reference		0.69 (0.34–1.42)	0.309
Yes	42 (60.9%)	27 39.1(%)	1.03 (0.58–1.82)	0.934		
**Smoking**						
No	98 (61.6%)	61 (38.4%)	Reference		1.16 (0.54–2.48)	0.706
Yes	45 (60.0%)	30 (40.0%)	1.07 (0.61–1.88)	0.811		
**LEEP**						
No	113 (60.4%)	74 (39.6%)	Reference		1.39 (0.50–3.83)	0.532
Yes	34 (66.7%)	17 (33.3%)	0.76 (0.40–1.47)	0.417		
**HPV Vaccination**						
No	58 (59.8%)	39 (40.2%)	Reference		0.68 (0.32–1.46)	0.330
Yes	69 (64.5%)	38 (35.5%)	0.82 (0.46–1.46)	0.490		
**Baseline HPV Group**						
Others	24 (55.8%)	19 (44.2%)	Reference		1.38 (0.56–3.43)	0.484
HPV 16,18 and Other High-Risk Types	124 (63.3%)	72 (36.7%)	0.73 (0.38–1.42)	0.363		

## Data Availability

The data presented in this study are not publicly available due to ethical and privacy restrictions related to patient confidentiality. Anonymized data supporting the findings of this study are available from the corresponding author upon reasonable request and with permission from the relevant institutional ethics committee.

## Abbreviations

AGC	Atypical glandular cells
aOR	Adjusted odds ratio
ASC-H	Atypical squamous cells—cannot exclude high-grade squamous intraepithelial lesion
ASC-US	Atypical squamous cells of undetermined significance
CI	Confidence interval
CIN	Cervical intraepithelial neoplasia
DNA	Deoxyribonucleic acid
EGCG	Epigallocatechin gallate
HPV	Human papillomavirus
hrHPV	High-risk human papillomavirus
HSIL	High-grade squamous intraepithelial lesion
LEEP	Loop electrosurgical excision procedure
LSIL	Low-grade squamous intraepithelial lesion
NILM	Negative for intraepithelial lesion or malignancy
OR	Odds ratio
SPSS	Statistical Package for the Social Sciences
WHO	World Health Organization
